# Children’s Coping in Disasters: Developments, Challenges, and Future Directions

**DOI:** 10.1007/s11920-025-01654-0

**Published:** 2025-12-08

**Authors:** Tara Powell, Flora Cohen, Maegan Ramchal

**Affiliations:** University of Illinois Urbana-Champaign School of Social Work, 1010 West Nevada, Urbana, IL 61801 USA

**Keywords:** Disaster, Children and youth mental health, Coping, Culture

## Abstract

**Purpose of Review:**

We review the published literature on children’s coping in disasters, focusing on disaster type, cultural and socio-demographic influences, and the methods used to assess coping.

**Recent Findings:**

Disasters affect children and youth in diverse ways, shaped by both disaster type and broader contextual factors. Research has grown considerably over the past two decades, offering insights into adaptive and maladaptive coping in disaster contexts. However, the field remains constrained by several limitations: a lack of consensus on how coping is conceptualized in children, limited standardized measures specific to disaster contexts, and few culturally adapted tools that capture diverse coping processes across settings.

**Summary:**

A deeper understanding of children’s disaster coping can be achieved by addressing gaps in definitions and tools through clearer conceptualization, culturally grounded measures, and longitudinal, multi-systems approaches. Addressing these gaps will help strengthen supports for children facing the increasing frequency and severity of disasters.

## Introduction

Over the past two decades, the frequency and severity of disasters, both manmade and climate-related, have more than doubled [1]. Globally, approximately 400 climate-related disasters occur each year [2]. Extreme weather events such as hurricanes, drought, floods, wildfires, and extreme temperatures have been coupled with geophysical events such as earthquakes and tsunamis to generate catastrophic consequences for communities [3–5]. Man-made disasters, such as war and technological failures, have also escalated. In 2023, global armed conflict reached a three-decade high [6], and between 2000 and 2021, 5,390 technological disasters were recorded worldwide [7]. Driven by climate change, urbanization, and global instability, the effects of disasters on communities are multifaceted, increasing rates of disaster-related morbidity and mortality, famine, displacement, and uncertainty [8, 9].

Children and youth are among the most vulnerable to the impacts of disasters. Nearly one billion children live in countries that are vulnerable to catastrophic consequences of climate disasters [10], and approximately 1 in 5 children globally live in conflict affected countries [11]. Disasters may cause young people to lose their homes and belongings, be separated from their families, or be seriously injured [12, 13]. After experiencing disasters, children and youth are at risk for myriad physical health challenges such as malnutrition, gastroenteritis, and asthma [14]. They also are at an increased risk of adverse mental health outcomes such as anxiety, depression, post-traumatic stress disorder (PTSD), and behavioral challenges [4, 15, 16].

Young people’s reactions and susceptibility to disasters varies due to biological, psychological, and contextual factors [17, 18]. Young children are especially vulnerable during disasters due to their developmental stage, often lacking the cognitive capacity to fully comprehend the experience and rely heavily on their caregivers for safety, comfort, and interpretation of events. This dependence can heighten their distress if caregivers are unavailable, overwhelmed, or themselves traumatized [19]. Although adolescents are less dependent on caregivers than younger children, they are undergoing critical stages of identity formation and emotional development. This stage of development—characterized by increasing autonomy and ongoing reliance on social and familial support—heightens adolescents’ vulnerability to disruptions in routine, peer relationships, and family stability [20].

Risk factors including displacement, family separation, or exposure to additional traumatic events can exacerbate emotional distress in young people [21]. Protective factors such as supportive relationships, stable routines, and access to mental health services can buffer the impacts of disasters and foster recovery [22]. The way a young person copes with a disaster also significantly shapes their recovery trajectory. However, inconsistencies in defining and conceptualizing coping often fail to account for the contextual, cultural, and developmental factors that shape coping processes in disaster contexts [23–25]. This review seeks to examine the recent literature about children’s coping in disasters. Specifically, we examine the literature from 2022 to 2025 on coping among children and youth in disasters, with attention to disaster type (i.e., climate related, biological, technological), cultural and socio-demographic factors, and limitations in measurement.

### Children and Youth Coping with Disasters

**Fig. 1:**
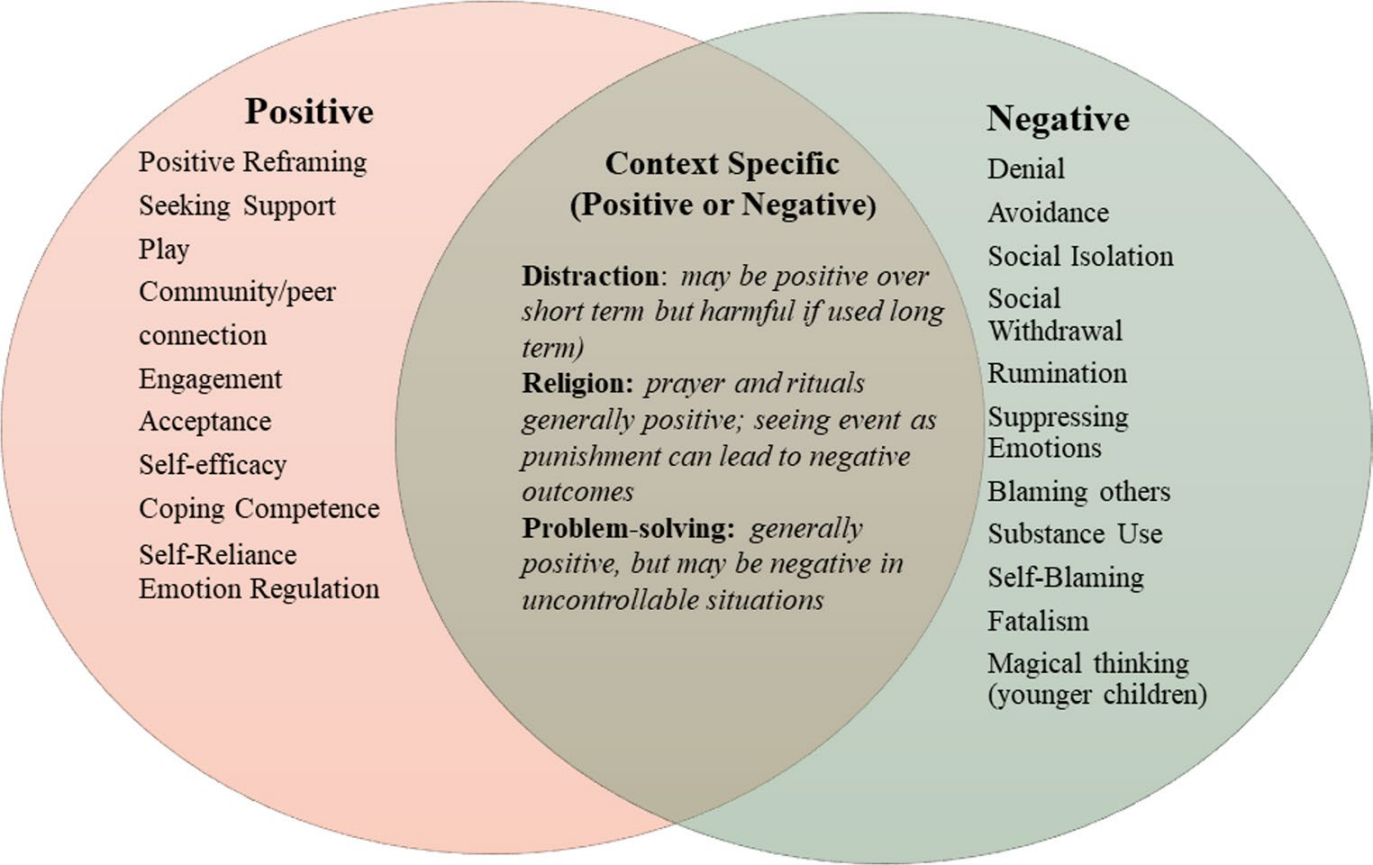
Coping strategies associated with positive or negative outcomes

The rise in climate and manmade disasters, alongside the COVID-19 pandemic has led to a substantial increase in research on young people’s coping during crises. The disaster literature consistently classifies coping into categories such as adaptive versus maladaptive or positive versus negative, linking these strategies to adjustment or maladjustment in children and youth [17, 25, 26]. Disaster scholarship emphasizes coping as a multidimensional construct, that can include internal (e.g., emotional regulation, cognitive restructuring, acceptance) and external (e.g., problem solving, seeking social support, disengagement) processes. This multidimensional approach captures both positive and negative strategies used by children [26, 27]. While children’s coping in disasters is recognized as both external and internal facing, terminology varies across studies. External coping, which has also been labeled as problem-focused, includes outwardly facing approaches to manage the stressor such as seeking support or taking action to address the concern. Internal coping, often described as emotion-focused, refers to self-directed responses such as re-framing, acceptance, or social isolation [28]. Nuances, however, exist in these classifications. For example, problem-solving can be both internal, where a child may write down healthy strategies to cope with the stressor, or external in which a child takes action to reduce the stressor [29]. Coping styles related to maladjustment (typically labeled as negative), include, but are not limited to, social withdrawal, avoidance, rumination, and blaming others [30]. Coping strategies related to fewer mental health symptoms generally include problem solving, positive re-framing, and support seeking [25, 30]. Figure 1 illustrates coping styles associated with positive and negative psychological outcomes.

### Coping by Disaster Type

The impact of disasters on children and youth varies, with consequences shaped by both the type of disaster and the broader context. Factors such as cause, frequency, controllability, speed of onset, geographic scope, disruptive potential, and likelihood of recurrence all influence outcomes for individuals and communities. While research suggests that children’s PTSD symptoms are more strongly predicted by the degree of exposure than by disaster type [31], the nature of the event still plays an important role in shaping coping and recovery. These event characteristics not only determine the challenges young people encounter but also influence the strategies they use to cope, whether turning inward to manage emotions or outward to seek solutions and support.

#### Climate Disasters

Rapid-onset climate-related disasters, such as tornadoes and tsunamis, strike suddenly and often cause catastrophic destruction, damaging infrastructure, displacing populations, and triggering significant economic loss [32]. In contrast, disasters like flooding and drought unfold over longer periods, creating prolonged disruptions that wear on families and communities [33]. Earthquakes, though sudden, may generate ongoing trauma through aftershocks, which serve as persistent reminders of the initial event and sustain heightened fear [34]. Since Hurricane Katrina struck the U.S. Gulf Coast in 2005, a substantial body of research has examined children’s coping in the aftermath of climate-related disasters. A 2023 meta-analysis by Raccanello and colleagues examined links between coping strategies and maladjustment, primarily in relation to PTSD symptoms following climate disasters. Maladaptive coping strategies—such as escape, social isolation, and opposition—were associated with poorer outcomes, while adaptive strategies like problem-solving and seeking social support predicted better adjustment [29].

#### Technological Disasters

Compared with climate-related disasters, the coping processes associated with technological disasters remain understudied. These events, which may intersect with climate disasters, often erode trust in infrastructure and social systems. For example, the devastation of Hurricane Katrina was compounded by the failure of levees meant to prevent flooding. Other technological disasters include oil spills, industrial fires, and nuclear accidents. Emerging literature, such as studies following the 2020 Beirut port explosion, have focused largely on diagnostic outcomes like PTSD, anxiety, and depression, with less attention to coping strategies tied to community cohesion and faith in institutions [35]. Notably, human-induced or human-precipitated disasters appear to generate longer-lasting psychological and social consequences, partly because they disrupt the fundamental trust between communities and the systems designed to protect them.

#### Biological Disasters

Biological disasters, such as the COVID-19 pandemic exposed young people to a range of adverse experiences, including parental loss, social isolation, service disruptions, and widening inequities [36]. Research consistently documents elevated symptoms of depression and anxiety, exacerbated by widespread childcare and school closures—63% of U.S. childcare centers shut down temporarily or permanently, and in many countries, schools remained closed for months or years [37]. These prolonged disruptions illustrate how pandemics differ from other disasters in their scale, duration, and ability to penetrate nearly every aspect of daily life. In response, researchers have examined children’s coping during the pandemic, revealing that negative strategies such as behavioral avoidance, denial, self-blame, substance use, venting, were consistently associated with heightened distress [38, 39]. Internalizing tendencies, including self-criticism and resignation, combined with loneliness and excessive internet use, further undermined mental health [40, 41]. By contrast, positive coping approaches such as problem-solving, cognitive restructuring, maintaining routines, physical activity, and prioritizing sleep supported better adjustment [41, 42]. Peer connection, particularly through supportive social media interactions, emerged as an especially effective means of emotional regulation, while humor and constructive distraction (e.g., schoolwork, limited leisure screen time) also contributed to resilience [40].

#### Complex Emergencies

Complex emergencies such as armed conflict and political instability impact children and youth directly and indirectly through long-lasting and compounding stressors, including physical health impacts (e.g., malnutrition, communicable diseases, etc.), displacement, forced separation, and trauma exposure [43]. Studies have illustrated that children and youth who experience protracted crises such as war and armed conflict exhibit higher levels of psychological distress, including PTSD, depression, and anxiety compared to those exposed to other types of disasters [43, 44]. These conditions make coping a critical factor in understanding youth mental-health outcomes in crisis settings.

A study examining children exposed to Yemen’s armed conflict in Southern Saudia Arabia found that those with higher PTSD symptoms relied on avoidance and distraction based coping strategies more often than children with fewer symptoms [45]. For children experiencing prolonged violence in Syria, including mass displacement and recurrent trauma, adaptive coping strategies included reliance on family and support systems (Nisa, 2024). Importantly, for children living through prolonged armed conflict, strategies that appear maladaptive may serve as adaptive responses in the moment—particularly when more protective forms of coping, such as consistent family support, are not available. Children who rely on avoidant coping, for example, have stated that these strategies provide a sense of hope during an ongoing conflict (Nisa, 2024).

#### Climate Change

Unlike discrete disasters, climate change poses an ongoing and existential threat that generates collective fear and worry. Children are particularly vulnerable to “eco-anxiety,” the distress linked to awareness of climate change. In a 2021 survey of 10,000 young people (ages 16–25) across 10 countries, 84% reported being worried about climate change, with nearly two-thirds believing their governments had failed to protect them [46]. Feelings of helplessness, fear, and grief are intensified by family stressors, economic instability, cultural beliefs, and social influences [47]. Emerging qualitative research has found that coping strategies that help children cope with eco-anxiety tends to include problem-focused strategies such as taking action to manage climate related stressors or seeking information. Emotion-focused strategies include avoidance, or de-emphasizing the threat of climate change [48]. Léger-Goodes and colleagues [48] also introduced meaning-focused coping strategies which include acknowledging the stressor, while also participating in cognitive re-framing such as experiencing hope in relation to climate change.

### Culture and Coping

It is important to acknowledge cultural and contextual factors that shape coping, as children’s recovery strategies are deeply influenced by the values, beliefs, and social structures of their communities [44]. In collectivist cultures, children often rely more heavily on communal and family-based support, whereas in more individualistic contexts coping may blend family support with greater emphasis on personal agency. Lomeli-Rodrigues and colleagues [49], found that adolescent survivors of an earthquake and tsunami in Indonesia turned to collective activities such as playing with peers, connecting socially, and engaging in religious practices as central coping strategies. In countries such as Iran, Haiti, Sri Lanka, and Indonesia, disasters are sometimes interpreted as divine retribution, leading children and families to cope through prayer, ritual, or communal practices that simultaneously offer meaning and reinforce social bonds [44, 50]. Religious coping, however, illustrates the dual nature of coping: it can be protective when it provides comfort, meaning, and community, but harmful when framed as punishment or exclusion, which may intensify distress [44]. In Indigenous traditions, such as Native American folklore, lessons about animal behavior before storms have historically functioned as both explanatory narratives of disasters and coping tools, guiding communities in preparedness and collective response. Beyond religion and folklore, culturally specific coping strategies may include group singing, play, cultural bonding, and prioritizing family interdependence [25].

### Socio-Demographic Characteristics and Coping

Socio-demographic characteristics play a significant role in shaping how children and adolescents cope with disasters. Developmental stage is central, as young people learn and adopt coping mechanisms from the environments in which they are raised, often modeling strategies used by parents or caregivers [36]. Yet, when disasters undermine adults’ caregiving capacity, children may be left without adequate guidance to regulate emotions or engage in adaptive coping [51]. Although gender differences in coping behaviors among children remains underexplored, existing evidence suggests disparities in psychological outcomes, with boys and girls often exhibiting distinct patterns of post-disaster distress [50, 52]. Socioeconomic conditions also shape coping opportunities, as children with access to stable housing, education, and health services are better positioned to employ constructive coping strategies, while those lacking basic needs face heightened vulnerability and fewer adaptive options [53]. Finally, global disparities in infrastructure mean that children in lower-resource settings face additional challenges to recovery, as damage to schools, community networks, and health systems constrains the range of coping options available [53]. Despite its importance, the role of socio-demographic characteristics in children’s coping remains under-researched, underscoring the need for greater attention to demographic and economic factors that may influence children and youths coping after a disaster.

### Coping Measurement

Coping measurement among disaster-affected children and youth presents several challenges. First, many instruments assess either dispositional coping tendencies (“how you usually cope”) or event-specific responses, but disasters require measures that can capture coping as a process that shifts across phases, from immediate response to long-term recovery. For example, in a meta-analysis of coping among children and adolescents affected by natural disasters, Raccanello and colleagues [29] identified more than 15 coping measures, yet only one—the Event-Related Rumination Inventory—was specifically developed for children who have experienced a traumatic event. Similarly, Wolf and Schmitz’s [42], scoping review of child mental health during the COVID-19 pandemic, found that of the three coping measures used, only the Adolescent Social Connection & Coping during COVID-19 Questionnaire (ASC) was adapted to capture pandemic-specific coping experiences. While Armstrong and Potter’s [54] brief measure of pandemic-related coping marks a recent advancement in context-specific coping assessment, few additional tools have been developed.

### Synthesis of Findings and Recommendations to the Field

One of the primary challenges in disaster coping research is the lack of consensus on how coping is conceptualized and operationalized in children, compounded by the limited availability of standardized measures specifically designed for disaster contexts [30]. Due to inconsistent conceptualizations, validated and standardized coping measures for disaster contexts remain limited. Such variability perpetuates blurred boundaries between coping strategies, complicating efforts to build standardized assessments and impeding comparability of findings across studies [17, 55]. Second, existing tools often obscure conceptual boundaries by conflating coping strategies (e.g., seeking social support, distraction) with outcomes (e.g., resilience, emotion regulation), or by overlapping coping styles with symptoms (e.g., rumination items that resemble depressive symptoms), making interpretation difficult [56].

To advance the field, researchers should work toward a clearer and more unified conceptualization of coping in disaster contexts by establishing a consensus on definitions that distinguish coping strategies from outcomes and symptoms, ensuring conceptual clarity. This conceptualization would support the development of standardized, developmentally appropriate measures designed specifically for children. Collaborative, cross-disciplinary efforts, drawing from developmental psychology, social work, psychiatry, and education are needed to refine coping frameworks and produce validated tools that can be reliably applied across diverse disaster contexts.

A lack of culturally adapted and validated measurement tools also remains a consistent limitation in the disaster coping literature. Most measures were developed in Western contexts that focus on personal agency, whereas in collectivist or resource-limited settings, children may rely more heavily on family, community, spirituality, and cultural rituals—strategies that are often overlooked. Additionally, the majority of commonly used measures (e.g., Kidcope, CCSC, RSQ, Brief COPE, CERQ) are decades old, reflecting coping theories of the 1980s and 1990s rather than more contemporary understandings of trauma, resilience, and cultural nuances [57–61]. Few studies examine the influence of newer domains such as social media, despite its increasing role in how young people seek connection. These measures were also designed for general stress, illness, or clinical contexts rather than acute, large-scale disasters, therefore may fail to capture unique ways of coping with disaster-related stressors such as displacement, community loss, and chronic uncertainty. To address this gap, research should prioritize the development and validation of coping measures that are both culturally grounded and disaster specific. This includes adapting existing tools through participatory methods with children, families, and communities across contexts, as well as integrating newer domains of coping. Measures should also reflect more contemporary frameworks that capture the multi-systemic nature of coping across different disaster phases, rather than relying on outdated constructs developed for general stress or clinical settings.

Beyond the scarcity of disaster-specific tools, existing research on children’s coping in disasters faces additional limitations: most studies rely heavily on self-report measures of coping and coping self-efficacy, raising concerns about developmental appropriateness and reporting bias; there is no consensus on how best to culturally adapt coping instruments across diverse settings; and the majority of studies are cross-sectional, limiting insights into how coping evolves across the immediate, recovery, and long-term phases of disaster exposure. Research has also rarely accounted for the multiple systems including the family, school, community, and broader sociopolitical contexts that shape coping processes within and beyond the individual child. These gaps highlight the pressing need for more contextually sensitive, longitudinal, and developmentally appropriate approaches to assessing coping in disaster-affected children.

## Conclusion

Over the past few years, research on children’s coping in disasters has expanded considerably, offering valuable insights into how young people adapt to adversity. Yet, with the increasing frequency and severity of disasters, longstanding challenges remain. This is particularly evident in how coping is conceptualized, measured, and understood across diverse cultural and developmental contexts. Addressing these gaps through aligned definitions, culturally grounded disaster-specific tools, and longitudinal, multi-systems approaches is critical to advance the field of disaster research. Building on recent progress, the field is well positioned to generate more rigorous evidence that can strengthen supports for children navigating escalating global crises.

## Key References

- Kar, N. (2024). Coping strategies used by children and adolescents following disaster trauma: A review of associated factors and intervention options.*Odisha Journal of Psychiatry*,*20*(2), 43-51.
  - ○ This manuscript synthesizes evidence on coping strategies used by children and adolescents after disaster-related trauma and examines key factors shaping these responses. Kar highlights that problem-focused coping is generally protective, while avoidant and other maladaptive strategies are linked to greater psychological distress. The review also outlines interventions—such as cognitive–behavioral, psychosocial, and family-centered approaches—that strengthen adaptive coping. It underscores the need for developmentally and culturally tailored supports to enhance youths’ coping capacities in post-disaster contexts.
- Raccanello, D., Rocca, E., Barnaba, V., Vicentini, G., Hall, R., & Brondino, M. (2023). Coping strategies and psychological maladjustment/adjustment: A meta-analytic approach with children and adolescents exposed to natural disasters. *Child & Youth Care Forum.* 52 (1), New York: Springer US.
  - ○ This meta-analysis examines how coping strategies relate to psychological adjustment among children and adolescents exposed to natural disasters. Productive coping strategies are associated with better internalizing, externalizing, and overall adjustment outcomes, whereas avoidance- and emotion-focused coping correspond with greater maladjustment. Findings demonstrate that coping style is a significant determinant of post-disaster mental health and highlight the need for interventions that strengthen adaptive coping skills in young populations.
- Rahmani, M., Muzwagi, A., & Pumariega, A. J. (2022). Cultural factors in disaster response among diverse children and youth around the world. *Current psychiatry reports*, *24*(10), 481-491.
  - ○ This review explores how cultural beliefs, social norms, and community practices shape children’s and adolescents’ psychological responses to disasters. The authors show that culture influences trauma perceptions, coping preferences, help-seeking, and engagement with mental-health interventions. They note that culturally mismatched approaches can hinder recovery, especially in collectivist contexts or areas with limited mental-health infrastructure. The review emphasizes the need for culturally grounded, community-informed interventions that align with local values and strengthen family and social supports in post-disaster settings.
- Spencer, G., & Thompson, J. (2024). Children and young people’s perspectives on disasters–Mental health, agency and vulnerability: A scoping review. *International Journal of Disaster Risk Reduction*,*108*, 104495.
  - ○ This review synthesizes research on how children and young people understand and experience disasters, with attention to mental health, agency, and vulnerability. The authors find that youth report significant emotional and social disruption but also demonstrate meaningful agency in coping and decision-making. They highlight the limited incorporation of children’s perspectives in disaster research and policy and argue for more participatory, rights-based approaches to address structural vulnerabilities and strengthen child-centered preparedness and recovery.

## Data Availability

No datasets were generated or analysed during the current study.
